# Vaccine effectiveness against influenza A(H3N2) and B among laboratory‐confirmed, hospitalised older adults, Europe, 2017‐18: A season of B lineage mismatched to the trivalent vaccine

**DOI:** 10.1111/irv.12714

**Published:** 2020-02-05

**Authors:** Angela M. C. Rose, Esther Kissling, Alin Gherasim, Itziar Casado, Antonino Bella, Odile Launay, Mihaela Lazăr, Sierk Marbus, Monika Kuliese, Ritva Syrjänen, Ausenda Machado, Sanja Kurečić Filipović, Amparo Larrauri, Jesús Castilla, Valeria Alfonsi, Florence Galtier, Alina Ivanciuc, Adam Meijer, Aukse Mickiene, Niina Ikonen, Verónica Gómez, Zvjezdana Lovrić Makarić, Alain Moren, Marta Valenciano, I‐MOVE Hospital study team

**Affiliations:** ^1^ Epiconcept Paris France; ^2^ National Centre of Epidemiology CIBERESP Institute of Health Carlos III Madrid Spain; ^3^ Navarra Public Health Institute IdiSNA–CIBERESP Pamplona Spain; ^4^ Department of Infectious Diseases Istituto Superiore di Sanità Rome Italy; ^5^ Inserm F‐CRIN Innovative clinical research network in vaccinology (I‐REIVAC) Paris France; ^6^ CIC Cochin Pasteur université Paris Descartes Sorbonne Paris Cité hôpital Cochin AP-HP Paris France; ^7^ National Military–Medical Institute for Research and Development Bucharest Romania; ^8^ National Institute for Public Health and the Environment Bilthoven The Netherlands; ^9^ Department of Infectious diseases Lithuanian University of Health Sciences Kaunas Lithuania; ^10^ Finnish Institute for Health and Welfare Tampere Finland; ^11^ Instituto Nacional de Saúde Doutor Ricardo Jorge Lisbon Portugal; ^12^ Division for epidemiology of communicable diseases Croatian Institute of Public Health Zagreb Croatia; ^13^ CHU de Montpellier Inserm CIC 1411 Hôpital Saint‐Eloi Montpellier France; ^14^ Finnish Institute for Health and Welfare Helsinki Finland

**Keywords:** Europe, hospital, influenza, older adults, test‐negative design, vaccine effectiveness

## Abstract

**Background:**

Influenza A(H3N2), A(H1N1)pdm09 and B viruses co‐circulated in Europe in 2017‐18, predominated by influenza B. WHO‐recommended, trivalent vaccine components were lineage‐mismatched for B. The I‐MOVE hospital network measured 2017‐18 seasonal influenza vaccine effectiveness (IVE) against influenza A(H3N2) and B among hospitalised patients (≥65 years) in Europe.

**Methods:**

Following the same generic protocol for test‐negative design, hospital teams in nine countries swabbed patients ≥65 years with recent onset (≤7 days) severe acute respiratory infection (SARI), collecting information on demographics, vaccination status and underlying conditions. Cases were RT‐PCR positive for influenza A(H3N2) or B; controls: negative for any influenza. “Vaccinated” patients had SARI onset >14 days after vaccination. We measured pooled IVE against influenza, adjusted for study site, age, sex, onset date and chronic conditions.

**Results:**

We included 3483 patients: 376 influenza A(H3N2) and 928 B cases, and 2028 controls. Most (>99%) vaccinated patients received the B lineage‐mismatched trivalent vaccine. IVE against influenza A(H3N2) was 24% (95% CI: 2 to 40); 35% (95% CI: 6 to 55) in 65‐ to 79‐year‐olds and 14% (95% CI: −22 to 39) in ≥80‐year‐olds. Against influenza B, IVE was 30% (95% CI: 16 to 41); 37% (95% CI: 19 to 51) in 65‐ to 79‐year‐olds and 19% (95% CI: −7 to 38) in ≥80‐year‐olds.

**Conclusions:**

IVE against influenza B was similar to A(H3N2) in hospitalised older adults, despite trivalent vaccine and circulating B lineage mismatch, suggesting some cross‐protection. IVE was lower in those ≥80 than 65‐79 years. We reinforce the importance of influenza vaccination in older adults as, even with a poorly matched vaccine, it still protects one in three to four of this population from severe influenza.

## 1 INTRODUCTION

In Europe, most countries recommend seasonal influenza vaccination for populations at risk of severe disease, such as older adults (aged 65 years and above), or those with co‐morbid conditions like heart disease or diabetes.^1^ In February 2017, the World Health Organization (WHO) recommendations for the 2017‐18 trivalent influenza vaccine for the northern hemisphere were to include an A/Michigan/45/2015 (H1N1)pdm09‐like virus, an A/Hong Kong/4801/2014 (H3N2)‐like virus and a B/Brisbane/60/2008‐like virus (B/Victoria lineage). It was also recommended that quadrivalent vaccine contains the above three viruses and additionally a B/Phuket/3073/2013‐like virus (B/Yamagata lineage).^2^

The 2017‐18 influenza season in Europe saw co‐circulation overall of influenza A(H3N2), A(H1N1)pdm09 and B viruses, predominated by influenza B, although patterns of dominance varied across countries.^3^ Most (97%) of the B viruses typed were B/Yamagata.^3^ Most of the hospitalised severe acute respiratory illness (SARI) cases (occurring mainly in older adults) were caused by infection with influenza B.^3^

In February 2018, early influenza vaccine effectiveness (IVE) was estimated from five European studies, covering all ages and including both primary care and hospitalised patients. The IVE against influenza A(H3N2) from these studies across nine countries (including two multi‐country I‐MOVE/I‐MOVE+ studies) ranged from −42% to 7%, with IVE of 36%‐54% for influenza B and 55%‐68% for influenza A(H1N1)pdm09.^4^

The I‐MOVE (Influenza—Monitoring of Vaccines in Europe) hospital network has provided annual IVE for participating hospitals across Europe since 2015. In 2017‐18, we measured seasonal IVE against hospitalisation with influenza A(H3N2) and B among older adults (aged 65 years and over) in the European Union (EU) by age group, underlying co‐morbid conditions, and vaccination in previous seasons.

## 2 METHODS

### 2.1 Setting and population

In 2017‐18, the I‐MOVE hospital network included 23 hospitals from 10 study sites in nine countries across the EU (one site in each of Croatia, Finland, France, Italy, Lithuania, the Netherlands, Portugal, and Romania, and two study sites in Spain). The study population comprised all community‐dwelling elderly (≥65 years) admitted to these participating hospitals and diagnosed with SARI within the 7 days prior to swabbing. All study sites followed the same generic protocol, adapted to their setting.^5^ Following local requirements, each local protocol was submitted to and approved by the ethics committee(s) in each site. Patient consent was an inclusion criterion for the study in each hospital, described in the shared protocol.^5^

### 2.2 Definitions and exclusions

Study methods have been described in detail elsewhere.^6^ Briefly, this multi‐centre study used a test‐negative design (TND), in which cases were hospitalised SARI patients (ie with at least one systemic and one respiratory sign or symptom) with confirmed influenza A(H1N1)pdm09, A(H3N2) or B using reverse‐transcriptase polymerase chain reaction (RT‐PCR). Patients confirmed with influenza A(H1N1)pdm09 were excluded from analyses due to the small sample size within this population (N = 133). Controls were SARI patients who were influenza negative by RT‐PCR. The study period, defined in each site, was from the onset week of the first to the last confirmed case by influenza (sub)type. Patients were considered vaccinated if they had SARI onset >14 days after vaccination. Those with onset within the 14‐day period were excluded from analysis, and any patient vaccinated on or after SARI onset was recoded as unvaccinated. Any study site with <10 (sub)type‐specific cases was excluded from pooled analysis.

### 2.3 Analysis

We estimated overall adjusted IVE as IVE = (1‐OR_a_) × 100 using a one‐stage analysis of pooled individual data from all sites, for influenza A(H3N2) and B. We used logistic regression, with study site as a fixed effect. In secondary analyses, IVE for both subtypes was estimated and stratified by (a) age group (65‐79 years old and 80 years and over); (b) presence of at least two underlying chronic conditions (vs one or less); and (c) vaccination status over the current and previous two seasons, divided into five groups: vaccinated in this season only, vaccinated in either of the two previous seasons but not in this season, vaccinated in the current plus either of the previous two seasons, vaccinated in all three seasons, no vaccination in any of the three seasons the reference group). Penalised logistic regression was used where any stratum resulted in small enough numbers to lead to over‐fit models (defined as a having fewer than 10 cases or controls per model parameter).

For most analyses, the odds ratio (OR) was adjusted (OR_a_) by age, sex, symptom onset date, and presence of at least one, or the total number of underlying chronic conditions (none, one, two or more). The chronic conditions were selected from diabetes mellitus, cancer, heart/rheumatic/kidney/lung disease, being immunocompromised, or being obese. For each country, only chronic conditions for which vaccination was recommended were included. The OR_a_s for analyses stratified by individual chronic condition (where the total number of cases or controls with the condition was at least 50) were adjusted as stated above, but not by chronic condition. The variables onset date and age were modelled as continuous (using a restricted cubic spline with 3, 4 or 5 knots) or categorical terms, the choice of which for each model was determined by sample size and the Akaike's information criterion. The categorical term for onset date, “phase,” was determined for each site prior to pooling the data and comprised three categories: early (onset dates comprising up to and including the first 10% of cases), peak (representing 80% of cases) and late (for the final 10% of cases, with the latest onset date). The categorical term for age, “age group,” comprised two categories: 65‐79, and 80 years and over.

Sensitivity analyses included estimating IVE for those swabbed within 3 days and for those who were not on antivirals before swabbing.

## 3 RESULTS

**Figure 1:**
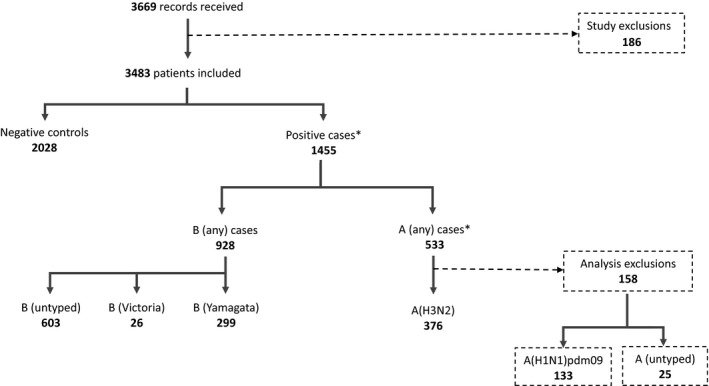
Flow chart of inclusions to the I‐MOVE hospital study Europe, influenza season 2017‐18 ^*^Note: there were three co-infected A(H1N1)pdm09 and B virus samples, three co-infected A (untyped) and B, and one co-infected A(H3N2) and A(H1N1)pdm09.

**Figure 2:**
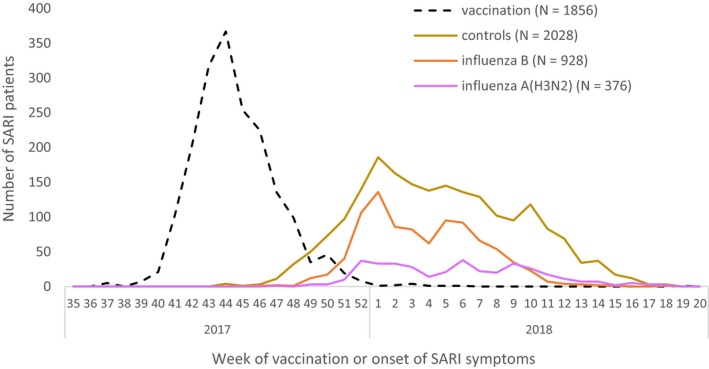
Week of vaccination or onset of severe acute respiratory infection (SARI) symptoms for SARI patients included in the I‐MOVE hospital study, Europe, influenza season 2017‐18

Data were received for 3669 patients in the 10 sites. After exclusions, 3483 patients were included in the study. Of these, 1455 were confirmed positive (any influenza) cases (42%), while 2028 were negative controls. There were 533 influenza A cases, of which 376 were confirmed as influenza A(H3N2) and 133 as influenza A(H1N1)pdm09 (25 untyped). Of the 928 influenza B, 299 were B/Yamagata, and 26 B/Victoria, with 603 having no lineage defined. There were six A and B virus co‐infections and one co‐infection for A(H3N2) and A(H1N1)pdm09 (Figure 1). Of the 1856 (53%) vaccinated patients, 1502 (81%) were vaccinated before week 47 2017, 7‐8 weeks prior to the peak in influenza onset (Figure 2).

### 3.1 Influenza A(H3N2)

Cases and controls were similar in terms of median age (81 vs 80 years), sex (52% vs 53% being male) and vaccine status (60% vs 62% vaccinated in the current and 61% vs 62% in the previous season) (Table 1). One vaccinated patient (<0.1%; a control) received quadrivalent, and the others received trivalent inactivated influenza vaccine. All vaccines were egg‐propagated. Over 90% of both cases and controls had at least one underlying chronic condition (*P* = .360), the main one of which was heart disease, with almost two‐thirds of both cases and controls affected (*P* = .283; Table 1). More controls than cases had lung disease (*P* = .027), but more cases than controls had rheumatic disease (*P* = .002; Table 1).

**Table 1:** Characteristics of influenza A(H3N2) and B hospitalised cases (n = 376 and 928) and test‐negative controls (n = 1530 and 1905) included in the I‐MOVE hospital study, Europe, influenza season 2017‐18*

	Influenza A(H3N2)		Influenza B	
	N = 376 cases/ total (%)	N = 1530 Test‐negative controls/Total (%)	N = 928 cases/ total (%)	N = 1905 Test‐negative controls/Total (%)
Median age in years (interquartile range)	81 (73‐86)	80 (73‐86)	78 (72‐84)	79 (72‐85)
Missing values	0	0	0	0
Characteristic				
Aged 65‐79 y	165/376 (43.9)	744/1530 (48.6)	528/928 (56.9)	**981/1905 (51.5)**
Male	193/375 (51.5)	810/1530 (52.9)	448/926 (48.4)	**1023/1905 (53.7)**
Vaccination status				
Seasonal influenza vaccination 2017‐18	224/376 (59.6)	952/1530 (62.2)	405/928 (43.6)	**1122/1905 (58.9)**
Seasonal influenza vaccination 2016‐17	225/372 (60.5)	946/1521 (62.2)	401/912 (44.0)	**1108/1878 (59.0)**
Underlying conditions				
Heart disease	242/373 (64.9)	941/1524 (61.7)	565/925 (61.1)	1216/1897 (64.1)
Lung disease	150/372 (40.3)	**712/1524 (46.7)**	335/928 (36.1)	**879/1897 (46.3)**
Diabetes	122/375 (32.5)	456/1527 (29.9)	270/928 (29.1)	568/1900 (29.9)
Rheumatic disease	85/373 (22.8)	**241/1520 (15.9)**	98/924 (10.6)	**275/1892 (14.5)**
Kidney disease	77/373 (20.6)	324/1524 (21.3)	156/926 (16.8)	**384/1898 (20.2)**
Cancer	75/376 (19.9)	339/1527 (22.2)	167/926 (18.0)	403/1901 (21.2)
Obesity	39/371 (10.5)	125/1521 (8.2)	110/923 (11.9)	184/1896 (9.7)
Immunocompromised	16/374 (4.3)	85/1518 (5.6)	34/925 (3.7)	88/1891 (4.7)
At least one underlying condition	336/376 (89.4)	1382/1529 (90.4)	822/928 (88.6)	**1729/1904 (90.8)**
At least two underlying conditions	247/376 (65.7)	1024/1529 (67.0)	530/928 (57.1)	**1272/1904 (66.8)**
Functional impairment	97/374 (25.9)	**548/1519 (36.1)**	318/922 (34.5)	665/1888 (35.2)
Hospitalisation in past year	186/374 (49.7)	783/1518 (51.6)	401/909 (44.1)	871/1850 (47.1)
Current smoking	58/239 (24.4)	347/1171 (29.7)	189/762 (24.9)	399/1530 (26.2)
Potential for misclassification				
Swabbing within 3 d of onset	232/376 (61.7)	933/1530 (61.0)	470/928 (50.6)	**1083/1905 (56.9)**
Received antivirals before swabbing	46/367 (12.5)	**60/1520 (3.9)**	144/912 (15.8)	**93/1895 (4.9)**
Study sites	Influenza A(H3N2)	Test‐negative controls	Influenza B	Test‐negative controls
Spain (Aragon and País Vasco regions)	155/376 (41.2)	539/1530 (35.2)	252/928 (27.2)	515/1905 (27.0)
Spain (Navarra region)	130/376 (34.6)	405/1530 (26.5)	196/928 (21.1)	366/1905 (19.2)
Finland	31/376 (8.2)	87/1530 (5.7)	14/928 (1.5)	91/1905 (4.8)
Netherlands	19/376 (5.1)	84/1530 (5.5)	63/928 (6.8)	105/1905 (5.5)
Portugal	18/376 (4.8)	67/1530 (4.4)	48/928 (5.2)	65/1905 (3.4)
France	10/376 (2.7)	233/1530 (15.2)	60/928 (6.5)	262/1905 (13.8)
Croatia	5/376 (1.3)	15/1530 (1.0)	23/928 (2.5)	16/1905 (0.8)
Lithuania	5/376 (1.3)	48/1530 (3.1)	74/928 (8.0)	53/1905 (2.8)
Italy	1/376 (0.3)	28/1530 (1.8)	90/928 (9.7)	351/1905 (18.4)
Romania	2/376 (0.5)	24/1530 (1.6)	108/928 (11.6)	81/1905 (4.3)

* In bold type are those proportions of characteristics in controls for which Fisher's exact test was *P* < .05 when compared with cases.

The pooled adjusted IVE estimate against influenza A(H3N2) was 24%, with a 95% confidence interval (95% CI) of 2‐40%. Adjusted VE was 35% (95% CI: 6 to 55) in those aged 65‐79 years and 14% (95% CI: −22 to 39) in those ≥80 years. Patients with heart disease had an adjusted IVE of 39% (95% CI: 17 to 55) and those with lung disease had an IVE of 28% (95% CI: −5 to 51). Precision for IVE among patients with diabetes, cancer and kidney disease was very low (Table 2).

**Table 2:** Seasonal influenza vaccine effectiveness (IVE) against influenza A(H3N2) overall and stratified by patients’ characteristics, I‐MOVE hospital study, Europe, influenza season 2017‐18

Population/stratification characteristic	Vaccinated/cases	Vaccinated/controls	Adjusted IVE	95% CI
Aged 65 y and above—age/time^a^	219/359	922/1413	24	(2;40)
Aged 65 y and above—full model^b^	219/359	922/1413	24	(2;40)
Aged 65‐79 y^c^	86/155	422/672	35	(6;55)
Aged 80 y and above^c^	133/204	500/741	14	(−22;39)
By underlying disease				
One or no chronic conditions^d^	55/102	233/398	32	(−10;58)
Two or more chronic conditions^e^	165/258	689/1015	21	(−7;41)
Heart disease^e^	134/235	578/859	39	(17;55)
Lung disease^e^	89/149	454/677	28	(−5;51)
Diabetes^f^	84/121	291/429	‐5	(−67;33)
Cancer^e^	54/75	215/323	‐9	(−96;39)
Kidney disease^g^	51/74	199/307	‐13	(−104;37)
By previous vaccination^b^	N (cases)	N (controls)		
Unvaccinated in all three seasons	95	309	ref.	
Vaccinated in either prior season only	36	137	21	(−23;50)
Vaccinated current season only	14	51	22	(−50;59)
Vaccinated current and either prior season	30	119	37	(−2;61)
Vaccinated all three seasons	160	720	35	(12;52)
Sensitivity analyses				
Swabbed within 3 d^b^	133/222	568/876	29	(2;48)
Swabbed within 3 d, excluding day 0^h^	116/199	485/749	34	(8;53)
No antivirals before swabbing^i^	199/313	909/1388	17	(−9;36)

a Age/time: adjusted for study site, age (modelled as a linear term) and onset date (three phases: early, peak and late).

b Full model: adjusted as for age/time model, sex and presence of any chronic condition.

c Adjusted as for age/time model, sex and number of chronic conditions (modelled as a categorical variable: none, one, two or more).

d Adjusted for study site, sex, age (modelled as a linear term) and onset date (modelled as a categorical variable using onset month).

e Adjusted as for age/time model and sex.

f Adjusted for study site, sex, age (modelled as a linear term) and onset date (modelled using a restricted cubic spline [RCS] with 5 knots).

g Adjusted for study site, sex, age (modelled using a RCS with 3 knots) and onset date (modelled using a RCS with 5 knots).

h Adjusted for study site, sex, age (modelled using a RCS with 3 knots), onset date (three phases: early, peak and late) and presence of any chronic condition.

i Adjusted for study site, sex, age (modelled as a linear term), onset date (modelled using a RCS with 5 knots) and presence of any chronic condition.

Adjusted IVE against influenza A(H3N2) was 37% (95% CI: −2 to 61) among patients vaccinated in this and either of the two previous seasons, 35% (95% CI: 12 to 52) among patients vaccinated in all three seasons, 22% (95% CI: −50 to 59) for those vaccinated in the current season only and 21% (95% CI: −23 to 50) among those vaccinated in either of the two previous seasons but not in the current.

In the sensitivity analyses, IVE against influenza A(H3N2) was 29% (95% CI: 2 to 48) for those swabbed within 3 days and 17% (95% CI: −9 to 36) for those who were not on antivirals before swabbing.

### 3.2 Influenza B

Cases and controls were similar in terms of median age (78 vs 79 years). A higher proportion of controls than cases were male (56% vs 48%; *P* = .008) and vaccinated in this or the previous season (59% vs 44% for both seasons; *P* < .001) (Table 1). One vaccinated patient (<0.1%; a control) received quadrivalent influenza vaccine, and the rest received the lineage‐mismatched trivalent vaccine. All vaccines were egg‐propagated. Ninety‐three per cent of both cases and controls had at least one underlying chronic condition (*P* = .527), the main one of which was heart disease, with almost two‐thirds of both cases and controls affected (*P* = .124; Table 1). More controls than cases had lung disease (*P* < .001), rheumatic disease (*P* = .004) and kidney disease (*P* = .032; Table 1).

The pooled adjusted IVE for influenza B was 30% (95% CI: 16 to 41). For patients aged 65‐79 years, IVE was 37% (95% CI: 19 to 51) and in those ≥80 years, IVE was 19% (95% CI: −7 to 38). Patients with diabetes had an adjusted IVE of 48% (95% CI: 28 to 63), those with heart disease had an IVE of 30% (95% CI: 12 to 45%), and for lung disease patients, the IVE was 19% (95% CI: −6 to 39). Low precision was observed for IVE in patients with cancer and kidney disease (Table 3).

**Table 3:** Seasonal influenza vaccine effectiveness against influenza B overall and stratified by patient characteristics, I‐MOVE hospital study, Europe, influenza season 2017‐18

Population/stratification characteristic	Vaccinated/cases	Vaccinated/controls	Adjusted IVE	95% CI
Aged 65 y and above—age/time^a^	403/920	1121/1899	31	(17;43)
Aged 65 y and above—full model^b^	403/920	1121/1899	30	(16;41)
Aged 65‐79 y^c^	188/522	533/979	37	(19;51)
Aged 80 y and above^c^	215/398	588/920	19	(−7;38)
By underlying disease				
Fewer than two chronic conditions^d^	136/368	312/592	32	(7;50)
Two or more chronic conditions^d^	267/555	809/1310	29	(11;43)
Diabetes^e^	117/270	354/568	48	(28;63)
Heart disease^f^	248/564	723/1216	30	(12;45)
Cancer^f^	86/167	250/403	20	(−21;47)
Lung disease^g^	183/334	555/879	19	(−6;39)
Kidney disease^f^	85/156	237/384	4	(−48;38)
By previous vaccination^b^	N (cases)	N (controls)		
Unvaccinated in all three seasons	336	507	ref.	
Vaccinated in either prior season only	74	161	17	(−15;41)
Vaccinated current season only	30	73	25	(−20;53)
Vaccinated current and either prior season	41	118	41	(12;61)
Vaccinated all three seasons	304	882	35	(20;48)
Sensitivity analyses	Vaccinated/cases	Vaccinated/controls		
Swabbed within 3 d^b^	225/467	669/1081	33	(14;47)
Swabbed within 3 d, excluding day 0^h^	200/420	578/940	34	(15;49)
No antivirals before swabbing^h^	382/762	1096/1800	29	(14;41)

a Age/time model: adjusted for study site, age (modelled as a linear term) and onset date (modelled using a restricted cubic spline [RCS] with 5 knots).

b Full model: adjusted as for age/time model, sex and number of chronic diseases (none, one, two or more).

c Adjusted for study site, sex, age (modelled as a linear term), onset date (modelled using a RCS with 5 knots) and presence of any chronic diseases for the older age group, but number of chronic diseases (none, one, two or more) for the younger age group.

d Adjusted for study site, sex, age (modelled as a linear term), onset date (modelled using a RCS with 5 knots) and number of chronic diseases (none, one, two or more).

e Adjusted for study site, sex, age and onset date (age and onset date modelled using a RCS with 4 and 5 knots, respectively).

f Adjusted for study site, sex, age (modelled as a linear term) and onset date (modelled using a RCS with 5 knots).

g Adjusted for study site, sex, age and onset date (age and onset date modelled using a RCS with 3 and 5 knots, respectively).

h Adjusted for study site, sex, presence of any chronic diseases, age and onset date (age and onset date modelled using a RCS with 4 and 5 knots, respectively).

Adjusted VE against influenza B was 41% (95% CI: 12 to 61) for those vaccinated in both the current and either of the two prior seasons, 35% (95% CI: 20 to 48) for those vaccinated in all three seasons, 25% (95% CI: −20 to 54) for those only vaccinated in this season and 17% (95% CI: −16 to 41) for those vaccinated only in one of the two previous seasons.

In sensitivity analyses, IVE against influenza B was 33% (95% CI: 14 to 47) for those swabbed within 3 days and 29% (95% CI: 14 to 41) for those who were not on antivirals before swabbing.

## 4 DISCUSSION

Our results suggest VE against influenza A(H3N2) and B of <31% in older hospitalised adults, with lower VE point estimates in those aged 80 years and over (<20%) than in those aged 65‐79 years (>34%), although 95% CIs were wide. Considering previous vaccination over this and the prior two seasons (2015‐16 and 2016‐17), a similar pattern was suggested for both influenza A(H3N2) and B, with IVE point estimates being higher among patients who had been vaccinated in the current season and both or either of the previous two, than for those vaccinated only in the current season, suggesting boosting of residual protection from previous vaccination (although 95% CIs were wide).

Adjusted IVE against hospitalised influenza A(H3N2) among the elderly in the European region was 24%, only slightly higher than that found in the previous 2016‐17 season (17%).^7^ Our estimate was in line with other estimates globally for the 2017‐18 season, most of which were below 50%,^8^, ^9^ as they have been for almost a decade.^10^, ^11^, ^12^, ^13^, ^14^, ^15^, ^16^, ^17^ A recent systematic review of studies using the test‐negative design estimated a pooled IVE of 24% (95% CI: −6 to 45) against influenza A(H3N2) in older adults (>60 years) for the consecutive seasons 2004‐2015.^18^ Importantly, even when the vaccine matches the circulating viral strain for influenza A(H3N2), adaptations of the vaccine viruses to propagation in eggs can result in antigenic changes. The adaptation of a loss of glycosylation site on the influenza haemagglutinin, for example, was hypothesised to contribute to the poor VE against influenza A(H3N2) seen in the 2014‐15 and 2016‐17 seasons.^19^

Against influenza B, despite a lineage mismatch in the trivalent vaccine component this season (B/Yamagata in circulation vs B/Victoria in the trivalent vaccine), IVE was 30%, implying the possibility of cross‐protection.^20^ This IVE is lower, however, than the 52% seen in 2015‐16, when there was also a lineage mismatch for B (albeit the other way around, with B/Yamagata in the vaccine and B/Victoria in circulation).^21^, ^22^ Colleagues in Canada and Spain studying all age groups in an outpatient setting found a slightly higher IVE against influenza B than we did for the 2017‐18 season (39%; 95% CI: 23 to 52 and 41%; 95% CI: 20 to 56, respectively),^23^, ^24^ while another Spanish study with the same inpatient setting but in those >60 years found a similar result to ours (26%; 95% CI: −9 to 49).^25^

Older adults (aged 65 years and above) are known to be at increased risk of severe influenza requiring hospitalisation, especially those with underlying co‐morbid conditions.^26^ Some of these conditions (such as cancer, diabetes, heart and lung diseases) are more prevalent in this age group, in particular cardiovascular disease, the most common chronic condition to affect older adults with influenza.^27^ This was seen in our study, with almost two‐thirds of patients with either type of influenza, as well as test‐negative controls, having heart disease (Table 1), while the prevalence of each of the other co‐morbid conditions measured was below 50%.

Vaccination against influenza has been described as having low or very low effectiveness among the older adult population,^28^ as well as among patients with these co‐morbid conditions.^29^, ^30^, ^31^, ^32^, ^33^ Yet, influenza vaccination is considered the best available public health intervention to protect against influenza infection, and there is evidence of reduction in severe outcomes from influenza (hospitalisation^8^, ^34^, ^35^ or mortality^36^) in older adults, particularly for those with chronic co‐morbidities. Our results for the 2017‐18 season suggest vaccine protection from severe influenza A(H3N2) for about one in four hospitalised older adults, and for about two in five of these patients with heart disease. Protection from severe influenza B was seen for almost one in three hospitalised older adults (similar to the protection observed for those with heart disease), and for almost one in two of those with diabetes.

Different studies have had inconsistent results for the effect of previous vaccination on IVE, reflected in the recently published review by Ramsay et al.^37^ McLean et al's^38^ study looking at eight seasons of IVE in one population in the United States showed that IVE was similar for those vaccinated in the current season only, or the previous season only, or in both seasons, for both influenza A(H3N2) and B, and was highest in those whose vaccination history was unvaccinated during the previous 5 years. Martínez‐Baz et al^39^ found protection against influenza A(H3N2) and B for vaccination received in prior seasons. Rondy et al^40^ found that regardless of previous season vaccination, current season vaccination offered some protection against influenza A(H3N2) and B for hospitalised older adults in Europe. For European primary care patients, Valenciano et al^13^ detected no apparent pattern for the effect of previous vaccination on IVE. The antigenic distance (AD) hypothesis anticipates interference with current vaccine effect by prior season vaccination when two conditions for AD are both fulfilled: (a) AD is small between the two vaccines; and (b) AD is large between the prior vaccine and the current circulating strain.^41^ Based on circulating strains this season^42^ and recommended vaccines for this and the previous two seasons,^2^, ^43^, ^44^ the AD hypothesis would only be fulfilled for influenza B, and only for those vaccinated in 2016‐17. The expectation would thus be that there would be some interference from prior vaccination, at least for patients with influenza B. In contrast, in our study VE was higher for those vaccinated in the current season (for both influenza types) if they were also vaccinated previously.

Our study was limited by small sample size, particularly in patients with influenza A(H3N2), resulting in lower precision around the point estimates. A potential limitation was the low proportion (35%) of influenza B cases with lineage results in our study. However, this was obviated by national surveillance data from participating countries (where the overall proportion of influenza B cases with lineage was 56%; data not shown), as well as data from *Flu News Europe* (where the proportion of influenza B cases from sentinel sites across the EU with lineage was 48%^3^), both of which confirmed that 95% or more of influenza B cases with lineage were B\Yamagata. The benefits of this study are in the use of the same protocol by all study sites, the low numbers of missing data for all variables (see Table 1), and the study design, which ensures that all patients hospitalised in participating sites with influenza symptoms are included and tested. Only laboratory‐confirmed patients are classified as cases, thus eliminating misclassification bias. The sensitivity of PCR may decrease over time from symptom onset, but restricting analysis to cases swabbed within 3 days gave similar results, suggesting that misclassification was unlikely to have occurred.

## 5 CONCLUSION

For the 2017‐18 season among hospitalised older adults, IVE against influenza B was greater than that against A(H3N2), despite a trivalent vaccine and circulating B lineage mismatch, suggesting some cross‐protection (as quadrivalent vaccine was used in <0.5% of this population). Antigenic changes due to egg‐adaptation of the vaccine strain could have contributed to the low IVE against A(H3N2). Our results suggest lower IVE against both influenza A(H3N2) and B in those ≥80 years than in those aged 65‐79 years. We reinforce the importance of influenza vaccination in older adults as, even in seasons using trivalent vaccine with circulating influenza B lineage mismatch and adaption of the egg‐propagated vaccine virus, it remains preventive against severe influenza for at least one in four of this population.

## CONFLICT OF INTEREST

None declared. At the time of the study, Ritva Syrjänen was a co‐investigator in pneumococcal studies (not related to this study), for which the Finnish Institute for Health and Welfare has received research support from GlaxoSmithKline Biologicals.
