# Supplementary material for: Vaccine effectiveness against influenza A(H3N2) and B among laboratory‐confirmed, hospitalised older adults, Europe, 2017‐18: A season of B lineage mismatched to the trivalent vaccine
Source: Influenza Other Respir Viruses. 2020 Feb 5;14(3):302–10. doi: 10.1111/irv.12714 (PMC7182608; doi:10.1111/irv.12714)
Supplement: Supplementary file 1 [file IRV-14-302-s001.docx]

**Appendix 1. I-MOVE Hospital study team**

SPAIN

Alin Manuel Gherasim, Amparo Larrauri, Clara Mazagatos, National Centre of Epidemiology, Institute of Health Carlos III, CIBERESP, Madrid.

Francisco Pozo, Inmaculada Casas, National Centre for Microbiology, National Influenza Reference Laboratory, WHO-National Influenza Centre, Institute of Health Carlos III, Madrid.

Miriam García, Dirección General de Salud Pública, Aragón.

Miriam Latorre, María Isabel Millán Lou, Ana María Milagro Beamonte, Ana Martinez Sapiña, Hospital Universitario Miguel Servet, Zaragoza.

Madalen Oribe Amores, Subdirección de Salud Pública Gipuzkoa, País Vasco.

Amaia Aizpurúa, Gustavo Cilla, Milagrosa Montes, Laboratorio Hospital Donostia, CIBERER.

NAVARRA

Itziar Casado, Leticia Fernandino, Iván Martínez-Baz, Carlos Gómez Ibañez, Jesús Castilla, Instituto de Salud Pública de Navarra, IdiSNA, CIBERESP, Pamplona.

Carmen Martín, Ana Navascués, Aitziber Aguinaga, Carmen Ezpeleta, Complejo Hospitalario de Navarra, IdiSNA, Pamplona.

ITALY

Antonino Bella, Department of Infectious Diseases, National Institute of Health, Rome.

Valeria Alfonsi, Department of Infectious Diseases, National Institute of Health, Rome; Department of Medical–Surgical Sciences and Translational Medicine, University of Rome "Sapienza", Sant'Andrea Hospital, Rome.

Caterina Rizzo, Direction of Clinical Department, Bambino Gesù Children's Hospital, Rome.

Maria Rita Castrucci, Simona Puzelli, Department of Infectious Diseases, National Institute of Health, Rome.

FRANCE

F Galtier, Inserm, F-CRIN, Innovative clinical research network in vaccinology (I-REIVAC), Paris; CHU de Montpellier, Inserm CIC 1411, Hôpital Saint-Eloi, Montpellier.

C Merle, CHU de Montpellier, Inserm CIC 1411, Hôpital Saint-Eloi, Montpellier.

V Foulongne, CHU de Montpellier, Inserm CIC 1411, Hôpital Saint-Eloi, Montpellier.

C Agostini, CHU de Montpellier, Inserm CIC 1411, Hôpital Saint-Eloi, Montpellier.

P Géraud, CHU de Montpellier, Inserm CIC 1411, Hôpital Saint-Eloi, Montpellier.

L Crantelle, CHU de Montpellier, Inserm CIC 1411, Hôpital Saint-Eloi, Montpellier.

 F Lainé, CHU de Rennes, Inserm CIC1414, Hôpital Pontchaillou, Rennes.

G Lagathu, Service de Virologie, CHU de Rennes, Inserm CIC1414, Hôpital Pontchaillou, Rennes.

P Tattevin, Service de maladies infectieuses, CHU de Rennes, Inserm CIC1414, Hôpital Pontchaillou, Rennes.

S Jouneau, Service de Virologie, CHU de Rennes, Inserm CIC1414, Hôpital Pontchaillou, Rennes.

 O Launay, Université de Paris, Faculté de Médecine; AP-HP, Hôpital Cochin; Inserm, CIC Cochin-Pasteur, Paris.

M Lachatre, AP-HP, Hôpital Cochin; Inserm, CIC Cochin-Pasteur, Paris.

P Loubet, AP-HP, Hôpital Cochin; Inserm, CIC Cochin-Pasteur, Paris.

S Moncilovic, AP-HP, Hôpital Cochin; Inserm, CIC Cochin-Pasteur, Paris.

AS Lhonneur, Service de Virologie, AP-HP, Hôpital Cochin, Paris.

N Lenzi, I-REIVAC, CIC de Vaccinologie, Cochin-Pasteur, Paris.

 P Vanhems,I-REIVAC, Paris, Hôpital Edouard Herriot, Lyon.

S Amour, I-REIVAC, Paris, Hôpital Edouard Herriot, Lyon.

B Lina, I-REIVAC, Paris, Hôpital Edouard Herriot, Lyon.

ROMANIA

Mihaela Lazăr, Alina Ivanciuc, Daniela Pițigoi, Elena Stoian, Carmen Maria Cherciu, Maria Elena Mihai, “Cantacuzino” National Military–Medical Institute for Research and Development, Bucharest.

Bejean Codrina, Gabriela Juganariu, Plesca Claudia Elena, Teodor Andra, Elena Duca, Clinical Hospital of Infectious Diseases “Sf Parascheva”, Iasi.

Streinu Cercel Anca, Rodica Bacruban, National Institute for Infectious Diseases “Prof. Dr Matei Mals”, Bucharest.

Emanoil Ceausu, Simin-Aysel Florescu, Corneliu Petru Popescu, Grațiela Țârdei, Clinical Hospital of Infectious Diseases "Dr Victor Babes", Bucharest.

THE NETHERLANDS

Sierk Marbus, Adam Meijer, National Institute for Public Health and the Environment (RIVM), Bilthoven.

Peter Schneeberger, Florens Polderman, Tamara van Zuylen, Angela Bouman, Jeroen Bosch hospital, 's-Hertogenbosch.

Rianne van Gageldonk-Lafeber, Inge Roof, Sharon van den Brink, Gabriel Goderski, Lisa Wijsman, Pieter Overduin, National Institute for Public Health and the Environment (RIVM), Bilthoven

LITHUANIA

Monika Kuliese, Aukse Mickiene, Department of Infectious Diseases, University of Health Science, Kaunas.

A Bagdonas, Department of Internal Diseases, Kaunas Clinical Hospital, Kaunas.

G Damuleviciene, Department of Geriatrics, University of Health Sciences, Kaunas.

G Gefenaite, Department of Infectious Diseases, University of Health Sciences, Kaunas; Department of Health Sciences, Faculty of Medicine, Lund University, Lund, Sweden.

J Gudauskaite, Department of Geriatrics, University of Health Sciences, Kaunas.

K Krupeckaite, Department of Infectious Diseases, University of Health Sciences, Kaunas.

V Lesauskaite, Department of Geriatrics, University of Health Sciences, Kaunas.

L Jancoriene, Clinic of Infectious Diseases and Dermatovenerology, Institute of Clinical Medicine, Faculty of Medicine, Vilnius University, Vilnius; Centre of Infectious Diseases, Vilnius University Hospital Santaros klinikos, Vilnius.

B Zablockiene, Clinic of Infectious Diseases and Dermatovenerology, Institute of Clinical Medicine, Faculty of Medicine, Vilnius University, Vilnius; Centre of Infectious Diseases, Vilnius University Hospital Santaros klinikos, Vilnius.

FINLAND

Ritva Syrjänen, Niina Ikonen, Hanna Nohynek, Anu Haveri, Finnish Institute for Health and Welfare, Helsinki.

PORTUGAL

Ausenda Machado, Verónica Gómez, Ana Paula Rodrigues, Irina Kislaya, Baltazar Nunes, Patrícia Conde, Paula Cristóvão, Inês Costa, Pedro Pechirra, Raquel Guiomar, Liliana Dias, Rita Côrte-Real, José Poças, Paula Lopes and Maria João Peres, Departamento de Epidemiologia, Instituto Nacional de Saúde Dr. Ricardo Jorge, Lisbon, Portugal.

CROATIA

B. Kaic, S. Kurečić Filipović, V. Visekruna Vucina, I. Pem Novosel, Z. Lovrić Makarić, G. Petrovic, M.Zajec, Croatian Institute of Public Health, Division for epidemiology of communicable diseases, Zagreb.

V. Draženović, Croatian Institute of Public Health, Virology Department, Zagreb.

A. Vince, A. Topić, J. Budimir, E. Huljev, University Hospital for Infectious Diseases, Zagreb.

B. Lukšić, S. Karabuva, M. Čikeš, Clinical Hospital Centre Split.

EPICONCEPT

Angela MC Rose, Esther Kissling, Marc Rondy, Alain Moren, Marta Valenciano, Epiconcept, Paris, France.
